# Use of maximal dosage renin-angiotensin-aldosterone system inhibitors in a real life population of complicated type 2 diabetes – contraindications and opportunities

**DOI:** 10.1186/s12882-023-03205-2

**Published:** 2023-08-16

**Authors:** C. M. Gant, M. M. Oosterwijk, S. H. Binnenmars, G. J. Navis, H. Haverkate, S. J. L. Bakker, G. D. Laverman

**Affiliations:** 1 Department of Internal Medicine, Meander Medical Center, Amersfoort, The Netherlands; 2 Division of Nephrology, University Medical Center Groningen, University of Groningen, Groningen, The Netherlands; 3 Department of Nephrology and Hypertension, University Medical Centre Utrecht, Heidelberglaan 100, Utrecht, 3584CX The Netherlands; 4 Department of Internal Medicine, ZGT Hospital, Almelo, The Netherlands; 5 Hospital Pharmacy, ZGT Hospital, Almelo, The Netherlands; 6 Faculty of Electrical Engineering, Mathematics and Computer Science, University of Twente, Enschede, The Netherlands

**Keywords:** Type 2 diabetes, Cardiovascular disease, RAAS inhibition, Chronic kidney disease, Real world data

## Abstract

**Objective:**

Pharmacological inhibition of the renin-angiotensin-aldosterone-system (RAASi) is the cornerstone of hypertension treatment, renoprotection and secondary prevention of cardiovascular disease in patients with type 2 diabetes. Although there is a dose-dependent effect of RAASi with optimum protection when using maximal dose, little is known on actual use of maximal dosage RAASi in clinical practice. Here we investigate prevalence of maximal dosage RAASi, and contraindications for, optimizing RAASi dosage, in patients with complicated type 2 diabetes in a real-life clinical setting.

**Research design and methods:**

We performed a retrospective analysis in 668 patients included in the DIAbetes and LifEstyle Cohort Twente (DIALECT). We grouped patients according to no RAASi, submaximal RAASi and maximal RAASi use. All potassium and creatinine measurements between January 1st 2000 and date of inclusion in DIALECT were extracted from patients files. We identified determinants of maximal RAASi use vs. submaximal RAASi use with multivariate logistic regression analysis.

**Results:**

Mean age was 64 ± 10 years and 61% were men. In total, 460 patients (69%) used RAASi, and 30% used maximal RAASi. Maximal RAASi use was not statistically different between different indications for RAASi (i.e. hypertension, diabetic kidney disease, coronary heart disease and cerebrovascular disease; *P* > 0.05). Per patient, 2 [1-4] measurements of potassium and 20 [13–31] measurements of creatinine were retrieved, retrospective follow-up time was − 3.0 [-1.4 to -5.7] years. Pre-baseline hyperkalemia > 5.0 mmol/l and acute kidney injury were found in 151 (23%) patients and 119 patients (18%), respectively. Determinants of maximal RAASi were prior acute kidney injury (OR 0.51 (0.30–0.87)), increased albuminuria (OR 1.89 (1.17–3.08)) and total number of used antihypertensives (OR 1.66 (1.33–2.06)).

**Conclusions:**

Maximal dose RAASi is used in almost one third of complicated type 2 diabetes patients in a real-life setting. The prevalence of contraindications is considerable, but relative in nature, suggesting that it is worthwhile to explore strategies aimed at maximizing RAASi while circumventing the alleged contraindications.

**Supplementary Information:**

The online version contains supplementary material available at 10.1186/s12882-023-03205-2.

## Introduction

Renin-angiotensin aldosterone system inhibition (RAASi) is indicated for several conditions related to type 2 diabetes. Guidelines recommend RAASi as the first step treatment for hypertension in type 2 diabetes, for renoprotection in patients with diabetic kidney disease (DKD), and for secondary prevention in patients with established cardiovascular disease (CVD) [1–3]. However, real-life data on the actual use, and dosing, of RAASi in high-risk patients are scarce.

Studies have consistently demonstrated RAASi treatment improves all stages of albuminuria and reduces end-stage kidney disease, cardiovascular events and death [4–8]. The beneficial effects of RAASi are in part attributable to the blood-pressure lowering properties of RAASi, but RAASi also limits target organ damage development through blood pressure independent pathways, thereby further reducing risk of adverse renal events, cardiovascular events and all-cause mortality [9]. However, the ongoing high incidence of major adverse renal and cardiovascular events demonstrates the urgency to improve secondary prevention in at-risk patients.

To achieve optimum treatment effects of RAASi on blood pressure, albuminuria and target organ damage, it is vital that patients receive maximum effective dosage RAASi [10–14]. The dose-response curves of RAASi differ per desired effect. For instance, increasing RAASi from low to maximum dosage may not result in further blood pressure lowering [10]. However, for reducing albuminuria and for target organ protection, maximizing RAASi dose is of the utmost importance, as it reduces hard endpoints such as cardiovascular and all-cause mortality [10, 14]. In clinical practice, increasing RAASi dose may be hampered by adverse events such as hyperkalemia, acute kidney injury (AKI) and hypotension, therefore limiting renal and cardiovascular prevention [15].

So far, real-word data on the penetrance of the maximum dose RAASi prescription and contraindications for increasing RAASi dosage are scarce. Therefore, here we investigate use of, and documented contraindications for, maximum dosage RAASi in a real-life setting of type 2 diabetes patients.

## Methods

We performed a retrospective analysis using data from the DIAbetes and LifEstyle Cohort Twente (DIALECT). The study population and study procedures of DIALECT have been described previously [16]. DIALECT is an observational cohort study in patients with type 2 diabetes, which was designed to study lifestyle and pharmacological factors and their associations with clinical outcomes. The study has been approved by local institutional review boards (METC-Twente, NL57219.044.16; METC-Groningen, 1009.68020), is registered in the Netherlands Trial Register (NTR trial code 5855) and is performed according to the guidelines of good clinical practice and the declaration of Helsinki as revised in 2008. All participants signed an informed consent form before participation.

### Setting

Between September 2009 and May 2019, a total of 668 patients with type 2 diabetes were included in DIALECT [16]. DIALECT was performed in the outpatient clinic internal medicine of the Ziekenhuis Groep Twente (ZGT) Hospital, Almelo and Hengelo, the Netherlands. The ZGT Hospital is a secondary care center for diabetes treatment. In the Netherlands, referral criteria to secondary health care are as follows: inability to achieve adequate glycemic control with oral antidiabetic drugs or a standard insulin regimen, overt nephropathy (macroalbuminuria and/or estimated glomerular filtration rate (eGFR) below 60 ml/min/1.73m^2^), or multiple cardiovascular complications.

### Participants

All patients, aged 18 + years, visiting the internal medicine outpatient clinic for type 2 diabetes treatment were eligible for the study [16]. Exclusion criteria were inability to understand the informed consent procedure, insufficient command of the Dutch language, or renal replacement therapy. Eligible patients were selected from the electronic patient file and contacted by phone.

### Variables

At the clinic, sociodemographic characteristics, medical history, lifestyle behaviors, and current medications were registered and anthropometric dimensions were measured using standard procedures [16]. Medical history was additionally reviewed in the hospital electronic patient files independently by three different physician researchers. Medication use was extensively verified by data on drug prescriptions and drug deliveries provided by local pharmacies. In case of mismatching, the pharmacy delivery information was considered appropriate above the electronic patient file data, as pharmacy dispensing data more closely reflect the drugs the patient actually receives.

Coronary heart disease (CHD) was defined as the presence of one of the following items in medical history: physician diagnosed unstable angina pectoris, myocardial infarction, percutaneous coronary intervention, or coronary artery bypass graft. Cerebrovascular disease (TIA/CVA) was defined as a history of transient ischemic attack or cerebrovascular accident.

Blood pressure was measured in a supine position by an automated device (Dinamap®; GE Medical Systems, Milwaukee, WI) for 15 min with a 1 min interval. The mean systolic and diastolic pressure of the final three measurements was used for further analysis.

Blood was drawn from venipuncture, and morning void urine and 24-hour urine was collected as prescribed previously [16]. Data on dietary sodium intake was derived from the 24-hour urinary sodium excretion.

To study contraindications for maximal RAASi dosing we collected extensive retrospective data on serum potassium levels and creatinine levels from the hospital’s laboratory system. Potassium and creatinine data were extracted for all patients files using a query for all serum potassium and creatinine measurements registered between 01-01-2000 and date of inclusion in DIALECT.

### Targets and definitions

Patients were categorized in three groups according to RAAS inhibition use: patients who did not use RAASi (noRAASi), patients who used RAASi but not in the maximum dose (smRAASi) and patients who used maximal dose RAASi (mRAASi). Maximal daily dose of RAASi was based on existing literature, and is described in Additional file 1: Appendix 1.

As the use of direct renin inhibitors (i.e., aliskiren) and mineralocorticoid receptor antagonists (i.e., spironolactone and eplerenone) has not been well defined for renoprotection and cardiovascular prevention in patients with type 2 diabetes, we did not include these agents in the definition of maximal dosage RAASi.

Indications for RAASi were categorized as following: secondary prevention in patients with chronic kidney disease (i.e. albuminuria and/or eGFR < 60 ml/min/1.73m^2^), hypertension, stroke, heart failure, and CHD [1, 2].

Hyperkalemia was defined as serum potassium > 5.0 mmol/l, and serious hyperkalemia as potassium ≥ 6.0 mmol/l.

AKI was defined according to KDIGO guidelines: stage 1, rise in serum creatinine 1.5–1.9 times of previous value; stage 2, rise in serum creatinine of 2-2.9 times of previous value; stage 3, rise in serum creatinine 3 times or higher of previous value.

Moderately increased albuminuria was defined as > 30 mg/24 h albumin excretion, or increased albumin/creatinine ratio (> 2.5 mg/mmol for men and > 3.5 mg/mmol for women) in morning void urine when 24 h hour urine was missing. According to the KDIGO guidelines, target blood pressure was ≤ 140/90 mmHg for patients without albuminuria, and ≤ 130/80 mmHg for patients with albuminuria.

### Statistics

All statistical analyses were performed using Statistical Package for the Social Sciences (IBM, Chicago, IL, USA), version 22.0. Normality of data was assessed by visually inspecting the frequency histograms. Normally distributed data were presented as mean ± SD. Skewed variables were expressed as median [interquartile range]. Dichotomous variables were presented in number and percentage. Cases with missing data were excluded from the respective analyses.

Differences between the three RAASi groups (noRAASi, smRAASi, mRAASi) were tested using one-way analysis of variance with bonferroni post hoc analyses (normally distributed), Kruskal–Wallis (skewed), or χ2-test (categorical).

We investigated determinants of maximal RAASi vs. submaximal RAASi using multivariate logistic regression analyses. Candidates for the model were assessed using univariate logistic regression and literature research. The multivariate model was constructed using backward step analysis. For variables with high collinearity, one variable was chosen for inclusion in the multivariate model. Additionaly, we studied determinants of RAASi use versus no RAASi use using the same analysis.

## Results

Mean age of the 668 patients included in the study was 64 ± 10 years, 61% of patients were men, and median duration of type 2 diabetes was 12 [7-19] years (Table 1). Prevalence of co-morbidity was high as 35% (*n* = 231) of patients had albuminuria and 36% (*n* = 242) of patients had one or more macrovascular complications.

**Table 1:** Baseline characteristics of patients included in DIALECT, divided by dosage groups of renin-angiotensin-aldosterone system inhibition

		Total population	No RAASi	Submaximal RAASi	Maximum RAASi	*P*-value
	*n*	*n* = 668	*n* = 208 (31)	*n* = 262 (39)	*n* = 198 (30)	
Age years	668	64 ± 10	61 ± 12	65 ± 9	65 ± 9	< 0.001
Gender men, n (%)	668	405 (61)	131 (63)	147 (56)	127 (64)	0.15
Body mass index, kg/m2	666	32.6 ± 5.9	31.2 ± 5.9	33.1 ± 5.6	33.5 ± 6.1	< 0.001
SBP/DBP, mmHg	666/666	134/74 ± 16/10	140/78 ± 16/9	138/74 ± 16/10	141/76 ± 16/9	0.16/0.02
SBP < 100mmHg, n (%)	666	9 (1)	2 (1)	4 (2)	3 (2)	0.85
History of hypertension, n (%)	666	600 (89)	141 (68)	261 (100)	198 (100)	< 0.001
Heart frequency, beats/min	657	73 ± 13	73 ± 12	73 ± 13	72 ± 12	0.46
Smoker, yes/former (%)	668	106/345 (16/52)	43/106 (21/51)	38/126 (15/49)	25/113 (13/58)	0.05
Years since type 2 diabetes diagnosis, years	668	12 [7-19]	11 [5-17]	12 [7-19]	14 [8-20]	0.06
Serum HbA1c, mmol/mol	666	58 ± 12	57 ± 12	59 ± 13	59 ± 12	0.22
Insulin use, n (%)	668	427 (64)	125 (60)	175 (67)	127 (64)	0.32
Diabetic kidney disease, n (%)	664	314 (47)	70 (34)	127 (49)	117 (59)	< 0.001
eGFR < 60 ml/min/1.73m2, n (%)	668	176 (26)	40 (19)	72 (28)	64 (32)	0.01
eGFR < 30 ml/min/1.73m2, n (%)	667	25 (4)	7 (3)	11 (4)	7 (4)	0.88
Moderately increased albuminuria, n (%)	660	231 (35)	52 (25)	89 (34)	90 (46)	< 0.001
Retinopathy, n (%)	658	150 (23)	32 (16)	64 (25)	54 (27)	0.02
Polyneuropathy, n (%)	666	260 (39)	61 (30)	116 (44)	83 (42)	0.003
Coronary heart disease, n (%)	667	153 (23)	40 (19)	65 (25)	48 (24)	< 0.001
Cerebrovascular disease, n (%)	667	80 (12)	16 (8)	41 (16)	23 (12)	0.06
Heart failure						
Reduced ejection fraction, n (%)	433	13 (3)	0 (0)	7 (4)	6 (5)	0.16
Preserved ejection fraction, n (%)	433	11 (3)	2 (2)	6 (3)	3 (2)	
**Pharmacological management**						
Angiotensin converting enzyme inhibitors, n (%)	667	201 (30)	0 (0)	138 (53)	63 (32)	
Angiotensin II receptor blockers, n (%)	668	264 (40)	0 (0)	127 (49)	138 (70)	
Aldosterone antagonist, n (%)	668	64 (10)	16 (8)	22 (8)	26 (13)	0.13
Renin inhibitor, n (%)	668	6 (1)	5 (2)	1 (0)	0 (0)	0.02
Thiazide diuretics, n (%)	668	201 (30)	24 (12)	85 (32)	92 (47)	< 0.001
Loop diuretics, n (%)	668	121 (18)	19 (9)	65 (25)	37 (19)	< 0.001
Beta blockers, n (%)	668	317 (47)	69 (33)	128 (49)	120 (61)	< 0.001
Calcium antagonists, n (%)	668	171 (26)	31 (15)	56 (21)	84 (42)	< 0.001
Total number of antihypertensives	667	2 [1-3]	0 [0–2]	2 [2-3]	3 [2-4]	< 0.001
Blood pressure on target, n (%)	658	273 (42)	86 (42)	98 (38)	89 (46)	0.18
Hypertension requiring 4 + drugs, n (%)	657	173 (26)	7 (3)	74 (28)	92 (48)	< 0.001
**Serum biochemistry**						
Serum sodium, mmol/l	666	139 ± 3	139 ± 3	139 ± 3	139 ± 3	0.16
Serum potassium, mmol/l	667	4.1 ± 0.4	4.1 ± 0.4	4.1 ± 0.4	4.1 ± 0.5	0.74
Hyperkalemia, n (%)	667	12 (2)	3 (1)	5 (2)	4 (4)	0.90
**24-hour urine**						
Urinary sodium excretion, mmol/24 h	655	179 ± 77	171 ± 69	180 ± 78	185 ± 82	0.23
Sodium intake < 100 mmol/24 h	655	82 (13)	23 (11)	34 (13)	25 (13)	0.86
Urinary potassium excretion, mmol/24 h	656	76 ± 26	77 ± 28	75 ± 26	75 ± 24	0.51
**Hyperkalemia pre-baseline**						0.98
No, n (%)	668	517 (77)	162 (78)	202 (77)	153 (77)	
Yes, n (%)	668	151 (23)	46 (22)	60 (23)	45 (23)	
≥ 6.0 mmol/l	668	19 (3)	5 (2)	8 (3)	6 (3)	0.89
**AKI pre-baseline**						0.08
No, n (%)	668	542 (82)	169 (82)	202 (78)	171 (86)	
Yes, n (%)	668	119 (18)	36 (18)	56 (22)	27 (14)	
Stage 1, n (%)	668	98 (15)	31 (15)	45 (18)	22 (11)	
Stage 2, n (%)	668	19 (3)	3 (2)	13 (5)	3 (2)	
Stage 3, n (%)	668	10 (2)	3 (2)	5 (2)	2 (1)	

*SBP* Systolic blood pressure, *DBP *Diastolic blood pressure, *eGFR *Estimated glomerular filtration rate, *AKI *Acute kidney injury

In total, 460 (69%) of the patients used RAASi, and 198 (30%) of patients used RAASi in the maximal dosage. In general, patients on either smRAASi or mRAASi were older (65 ± 9 and 65 ± 9 years) than noRAASi (61 ± 12 years; *P* < 0.001) and had a higher body mass index (33.1 ± 5.6 and 33.5 ± 6.1 kg/m^2^) than noRAASi (31.2 ± 5.9 kg/m^2^; *P* < 0.001). The prevalence of diabetic kidney disease was highest in patients with maximum dosage RAASi (59% vs. 49% in smRAASi and 34% in noRAASi; *P* < 0.001). Prevalence of CHD (25% vs. 24%) and TIA/CVA (12 vs. 16%) did not materially differ between smRAASi and mRAASi. Interestingly, the median total number of used antihypertensive agents was highest in the mRAASi group (3 [2-4] vs. smRAAsi 2 [2-3] and noRAASi 0 [0–2]; *P* < 0.001). There was no difference in baseline serum potassium between the groups (*P* = 0.74). Urinary sodium excretion was not statistically significantly different between the groups (*P* = 0.23). In total, 13% of patients (*n* = 82) adhered to the guideline of < 100 mmol sodium intake per day (corresponding with < 6 gram sodium chloride), and this proportion did not differ between the groups (*p* = 0.86).

**Fig. 1:**
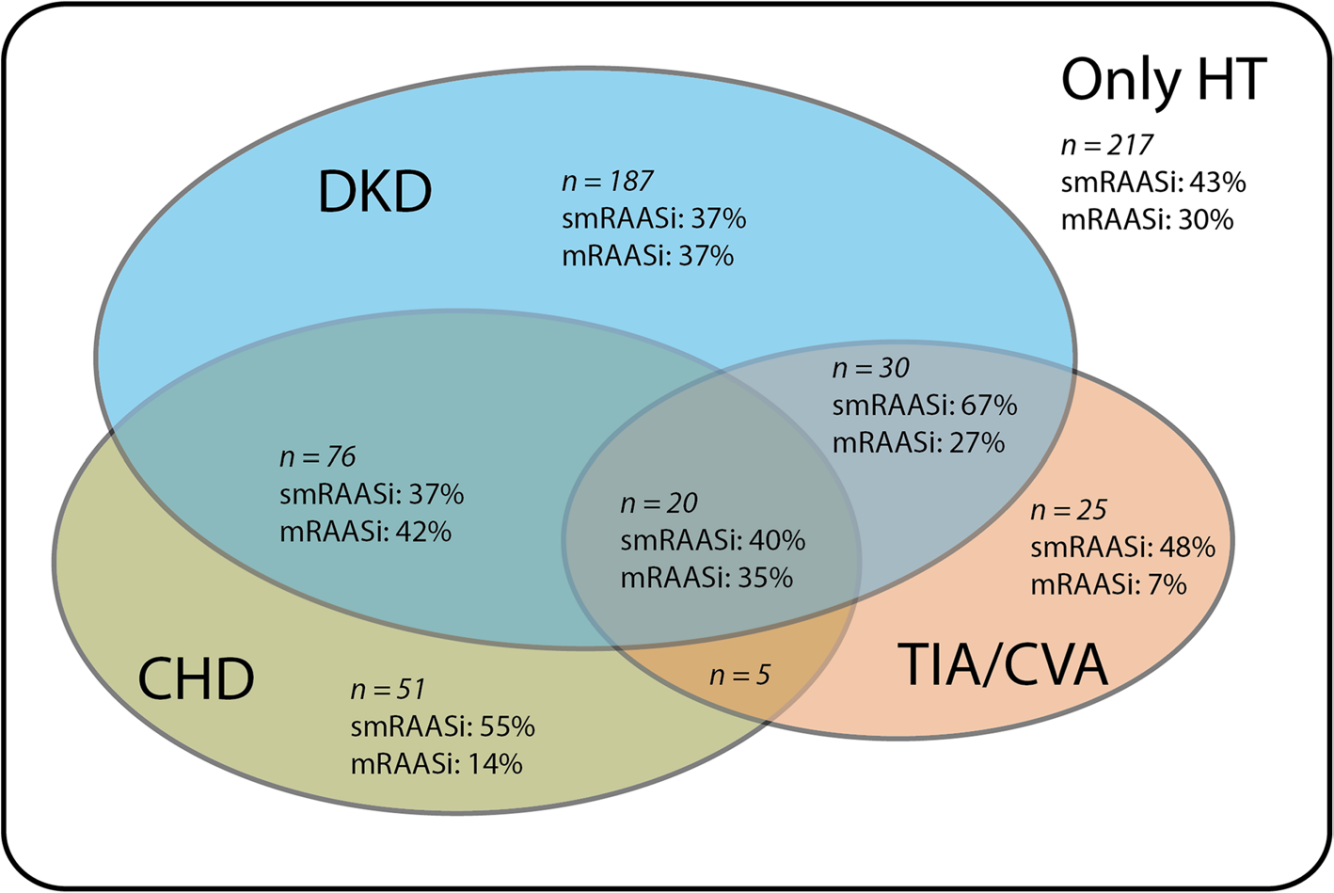
Proportion of patients using submaximal or maximal dosage of RAASi divided by indication Patients with hypertension, diabetic kidney disease and coronary heart disease most often used maximal dosage RAASi. DKD, diabetic kidney disease; CHD, coronary heart disease; TIA/CVA, cerebrovascular disease; HT, hypertension; smRAASi, use of submaximal dosage RAASi; mRAASi, use of maximal dosage RAASi

When investigating RAASi dosage per indication for RAASi (i.e., hypertension, DKD, CHD and TIA/CVA), we found that the proportion of patients on mRAASi was highest in those with the combination of hypertension, DKD and CHD (42%), although differences were not statistically significant (*P* > 0.05; see Fig. 1). There were no differences in smRAASi (4%) or mRAASi (5%) use in patients with heart failure with reduced ejection fraction (Table 1).

To investigate potential contraindications for mRAASi, we studied hyperkalemia and AKI that occurred before the baseline visit (Table 1). In total, there were 12,688 plasma potassium and 16,544 plasma creatinine measurements performed between 2000 and baseline assessments of the DIALECT cohort. Per individual patient, these were a median 2 [1-4] measurements of potassium and a median 20 [13–31] measurements of creatinine. Median retrospective follow-up time was − 3.0 [-1.4 to -5.7] years.

**Fig. 2:**
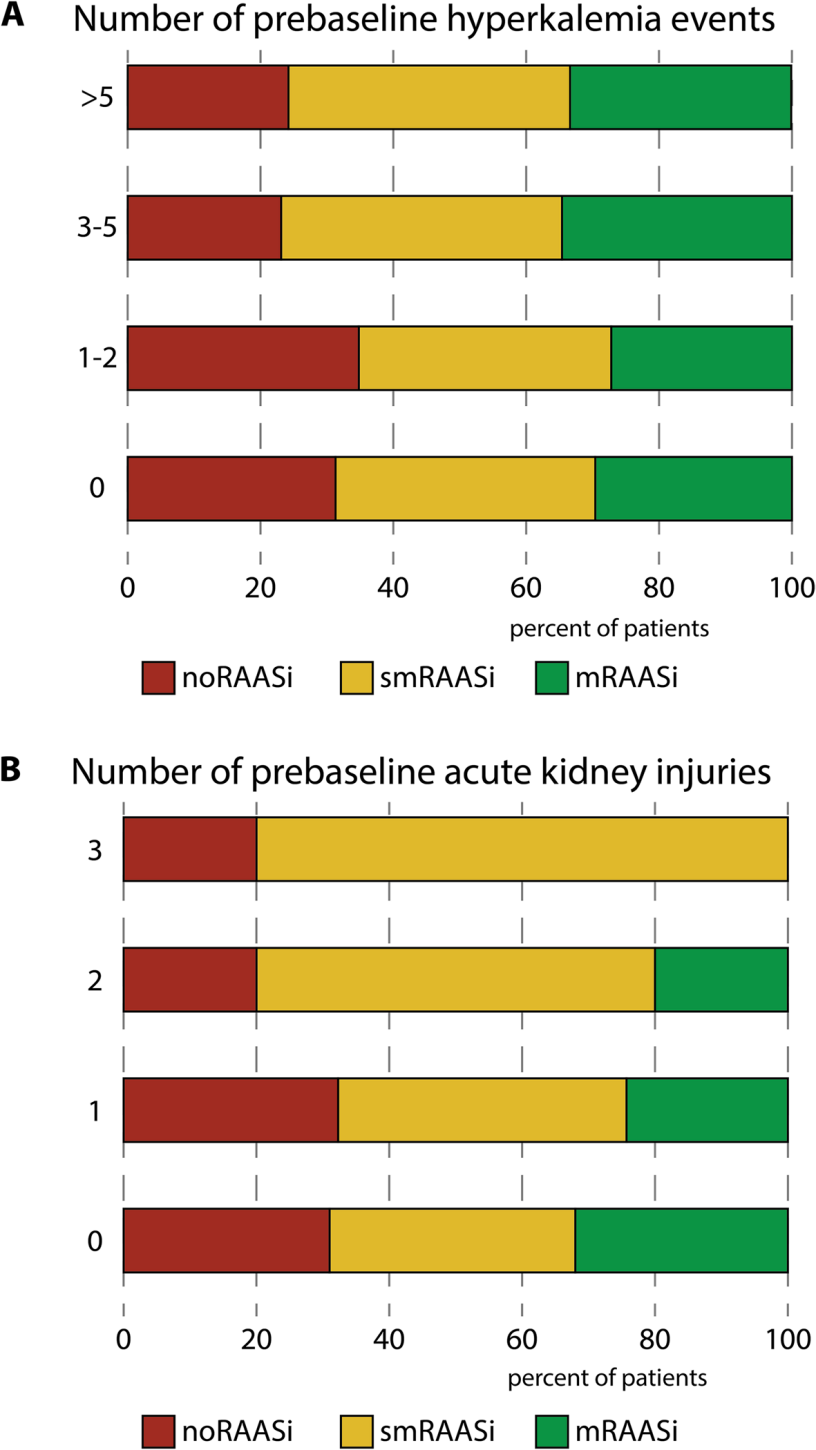
Use of maximal dosage RAASi divided by number of times (**A**) hyperkalemia and (**B**) acute kidney injury in pre-baseline measurements noRAASi, no use of renin-angiotensin-aldosteron system inhibition; smRAASi, use of submaximal dosage RAASi; mRAASi, use of maximal dosage RAASi

We found that 151 (23%) of patients had experienced hyperkalemia > 5.0 mmol/l at least once, 19 (3%) of whom at least once had a plasma potassium ≥ 6.0 mmol/l. Of the 33 patients with more than 5 previous hyperkalemic events, 8 were noRAASi (24%), 14 were smRAASi (42%) and 11 were mRAASi (33%; Fig. 2A). In patients with 1–2 or with 3 prebaseline hyperkalemic events, the distribution of noRAASi, smRAASi and mRAASi was not statistically different (*P* = 0.90). There were no differences in the prevalence of serious hyperkalemia ≥ 6.0 mmol/l between the RAASi categories (Table 1). At the time of the baseline visit, the actual prevalence of hyperkalemia was low: only 2% of the patients (*n* = 12) had a plasma potassium concentration > 5.0 mmol/l, the prevalence was not different in the RAASi groups.

An episode of AKI in the pre-baseline period was noted in 119 patients (18%), of which 10 patients (2%) had a stage 3 AKI. Further inspection of the electronic patient files demonstrated that none of the AKI events were related to initiation or dosage increase of RAASi. Of the 5 patients that had experience 3 pre-baseline AKIs, none used mRAASi, 4 used smRAASi, and 1 used noRAASi (Fig. 2B). There was a statistically non-significant trend towards lower use of mRAASi in those with 2 pre-baseline AKIs (20%), compared to mRAASi use in those with 1 (24%) or no (32%) prior AKIs (*P* = 0.17). At baseline, the prevalence of severe renal impairment (eGFR < 30 ml/min/1.73m^2^) as a potential contraindication was low (*n* = 25; 4%). Few patients had hypotension as a contraindication for increasing RAASi dosage (systolic blood pressure < 100 mmHg; *n* = 9; 1%).

To identify independent determinants of mRAASi compared to smRAASi, we performed univariate logistic regression analyses followed by multivariate logistic regression analysis. In univariate analysis (Table 2), the presence of moderately increased albuminuria is significantly associated with mRAASi (OR 1.67 (1.14–2.45)). Also, the total number of used antihypertensives was associated with mRAASi (OR 1.49 (1.26–1.77)). The number of prior AKIs was a negative predictor of mRAASi (OR 0.60 (0.40–0.89)). In multivariate analysis (Table 2), moderately increased albuminuria (OR 1.89 (1.17–3.08)), the total number of used antihypertensives (OR 1.66 (1.33–2.06)) and number of prior AKIs (OR 0.51 (0.30–0.87)) remained strong statistically independent predictors of mRAASi. Furthermore, TIA/CVA (OR 0.43 (0.21–0.90)) appeared as a negative predictor of mRAASi use. eGFR < 60 ml/min/1.73m^2^ or frequency of pre-baseline hyperkalemia were not associated with mRAASi compared to smRAASi.

**Table 2:** Logistic regression analysis on determinants of maximal RAASi vs. submaximal RAASi

	Univariate	Multivariate
	Exp(B) (95% CI)	
Age years	1.00 (0.98–1.03)	…
Gender, women vs. men	0.72 (0.49–1.04)	…
Body mass index, kg/m^2^	1.01 (0.98–1.05)	…
SBP, mmHg	1.01 (0.99–1.02)	…
DBP, mmHg	1.00 (0.99–1.02)	…
Heart frequency, beats/min	0.99 (0.98–1.01)	…
Smokers vs. non-smokers	0.93 (0.55–1.58)	…
Years since type 2 diabetes diagnosis, years	1.02 (1.00-1.04)	…
Serum HbA1c, mmol/mol	0.99 (0.98–1.01)	…
Insulin use	0.89 (0.60–1.31)	…
eGFR < 60 ml/min/1.73m^2^	0.79 (0.53–1.19)	…
Albuminuria	1.67 (1.14–2.45)	1.89 (1.17–3.08)
Retinopathy	1.14 (0.75–1.74)	…
Polyneuropathy	0.90 (0.62–1.31)	…
Coronary heart disease	0.97 (0.63–1.49)	…
Cerebrovascular disease	0.71 (0.41–1.22)	0.43 (0.21–0.90)
Total number of antihypertensives	1.49 (1.26–1.77)	1.66 (1.33–2.06)
Prebaseline hyperkalemia > 6 mmol/L	0.99 (0.34–2.91)	…
Number of prebaseline hyperkalemia events		
0	ref	…
1–2 times	0.96 (0.43–2.18)	…
3–5 times	0.91 (0.35–2.33)	…
> 5 times	1.04 (0.32–3.40)	…
Number of prebaseline acute kidney injury events	0.60 (0.40–0.89)	0.51 (0.30–0.87)

*SBP *systolic blood pressure, *DBP *diastolic blood pressure, *eGFR *estimated glomerular filtration rate

… excluded in the multivariate model in backward step analysis due to non-significance

In an additional analysis we investigated determinants of RAASi use versus no RAASi use, here we found that albuminuria or polyneuropathy were the strongest predictors of RAASi use (Supplementary Table 1). Prior AKI or hyperkalemia were not associated with the use of RAASi.

## Discussion

We investigated maximum dosage RAASi use in a real-life routine care setting of complicated type 2 diabetes patients. Only a third of these patients receive maximum dose treatment, which is in line with a previously reported proportion of 19–26%, found in a population of 195,327 patients with chronic kidney disease, diabetes and/or heart failure [15].

We examined possible causes for submaximal RAASi dosing. A considerable number of patients (23%) had a previous history of at least one measurement of hyperkalemia but this percentage was not different between the maximal (mRAASi) and submaximal (smRAASi) group. This, and the finding that the actual prevalence of hyperkalemia at baseline was 2%, suggests that other considerations must play a role, e.g., a clinical judgement of likelihood of reoccurrence. It should be emphasized however, that reducing the RAASi dose is not necessarily the primary option to prevent hyperkalemia. Reducing dietary potassium intake and/or the use of oral potassium binders are effective measures that should be taken into consideration as a first step in this respect.

Another potential factor limiting the use of RAASi is the occurrence of acute kidney injury (AKI) prior to baseline. We found that almost 20% of the patients had a history of AKI and that the occurrence of which was strongly associated with the use of submaximal dose RAASi instead of maximal dose. The occurrence of AKI, regardless of the cause, is often an important reason to pause RAASi to restore glomerular filtration pressure. However, in follow-up after AKI, persistent RAASi use is associated with decreased mortality, although the risk for hospitalization due to renal causes is higher [17]. As in our population, AKI prior to baseline did not occur after initiation or increasing dose of RAASi, and as current severe renal impairment eGFR < 30 ml/min/1.73m^2^ was rare (4% of patients), carefully monitored increase of RAASi dosage in patients with previous AKI could be attempted.

Finally, low blood pressure can be a limiting factor for maximal dosing of RAASi. We found that 1% of the patients had documented hypotension defined as a systolic blood pressure below 100 mmHg. Notably, the prevalence of difficult to treat hypertension was very high, as the majority of patients did not reach the target blood pressure (58%), and the median number of used antihypertensive agents was 2. When patients use an antihypertensive drug with limited renal and cardiovascular preventive effects, compared to RAASi, it could be considered to cease these agents, in order to initiate, or increase, RAASi therapy.

Taken together, there is ample opportunity to increase the dosage of RAASi in this population of patients with advanced type 2 diabetes, as (1) hyperkalemia occurs in a minority of patients and is manageable with alternative strategies; (2) hypotension is rare; (3) Previous AKI is relatively highly frequent and important to consider when maximizing RAASi treatment, but should not be a reason to forego RAASi optimization.

Because this was a retrospective and observational study, we cannot guarantee 100% correct adverse effects documentation in the patient files. On the other hand, to our knowledge, it is extremely rare that patients have a contraindication limiting the use of any RAASi, and if this were the case, it would be extremely unlikely that this would not be documented in the files.

Apart from contraindications, which are patient related factors, physicians may have preferences -in general and in specific situations- that are likely to influence the penetrance of maximal RAASi dosing. First, alternative antihypertensive drug classes could be preferred in certain situations due to competing indications, e.g., beta blockers or calcium channel blockers when CHD coincides with type 2 diabetes. Our finding that TIA/CVA was a negative predictor of mRAASi compared to smRAASi, could be a reflection of such a phenomenon. The guidelines on secondary stroke prevention recommend lowering blood pressure with combination therapy of multiple antihypertensives in a low dose, rather that maximizing RAASi [18]. Also, in hypertension treatment, an approach of combining drugs at submaximal doses has been advocated as a strategy to treat hypertension with less side-effects [19]. This strategy does not seem to be adopted here, as is indicated by our finding that the number of used antihypertensives was strongly positively associated with mRAASi.

The most important patient characteristics associated with mRAASi were the presence of moderately increased albuminuria, and difficult to treat hypertension. The former was an expected finding, as albuminuria is a very important indication for initiation of RAASi. However, even in patients with albuminuria, mRAASi use was still 46%, although ideally all patients should receive mRAASi, as it is associated with a decrease in hard cardiovascular and renal endpoints [11]. Our finding that difficult to treat hypertension is strongly associated with mRAASi suggests that clinicians often do increase RAASi dosage in the scope of blood pressure treatment. Surprisingly, coronary heart disease, which is a very important indication for RAASi, was not associated with mRAASi compared to smRAASi. Although these patients also use other agents which act on blood pressure and possibly renal function, our data demonstrated that the prevalence of unequivocal contraindications for increasing RAASi dosage is low, which suggests that there is enough possibility to increase RAASi dosage.

Reduction of dietary sodium intake is an additional well-established method to increase RAASi effectivity [20]. In our population, only 13% of patients adhered to the guideline on sodium intake of < 100 mmol/day. Therefore, next to increasing RAASi dosage, reducing dietary sodium could be a good strategy to increase RAASi efficacy.

This study has several strengths. First, it represents a real-life population of patients with complicated type 2 diabetes, and therefore provides important insights on implementation of pharmacological treatment in routine care. Also, our data on pharmacological drug use was extensively verified by review of the electronic patient files and by collection of drug delivery-data from local pharmacies. Furthermore, we extracted an ample amount of laboratory data on potassium and creatinine measurements prior to baseline to investigate the association between previous adverse events and current RAASi dosing.

This study also has some limitations. Because of the retrospective nature, the reasons not to choose maximum dose RAASi were not systematically registered in the patient files and neither was is information on side-effects. For the same reason, any relation between previous AKI or hyperkalemia and RAASi should be considered as hypothesis-generating. Additionaly, this was a single-center study performed in the Netherlands, therefore extrapolation to other populations should be done with caution.

In conclusion, we found that in a real-life population of patients with type 2 diabetes in routine clinical care, only a third of patients used maximum dosage RAASi. Although the prevalence of contraindications for maximal RAASi, such a hyperkalemia and AKI, is considerable, they are usually relative in nature. These findings suggest that, to improve renoprotection and cardiovascular risk management in type 2 diabetes, it is worthwhile to explore strategies aimed at maximizing RAASi while circumventing the alleged contraindication.

### Supplementary Information

**Additional file 1.**

## Data Availability

The datasets generated and/or analysed during the current study are not publicly available due to ongoing analyses, but are available from the corresponding author on reasonable request.
