# Supplementary material for: Use of maximal dosage renin-angiotensin-aldosterone system inhibitors in a real life population of complicated type 2 diabetes – contraindications and opportunities
Source: BMC Nephrol. 2023 Aug 16;24:240. doi: 10.1186/s12882-023-03205-2 (PMC10428595; doi:10.1186/s12882-023-03205-2)
Supplement: Supplementary file 1 — Additional file 1. [file 12882_2023_3205_MOESM1_ESM.docx]

**Online only supplementary material**

**Supplementary Table 1.** Logistic regression analysis on determinants of RAASi use versus no RAASi use in DIALECT.

|  |  | Univariate | Multivariate |
| --- | --- | --- | --- |
|  |  | Exp(B) (95% CI) |  |
| Age years | | 1.04 (1.02-1.05) | 1.03 (1.01-1.05) |
| Gender, women vs men | | 1.16 (0.82-1.62) | … |
| Body mass index, kg/m^2^ | | 1.07 (1.04-1.11) | 1.08 (1.04-1.12) |
| Heart frequency, beats/min | | 1.00 (0.99-1.01) | … |
| Smokers vs non-smokers | | 0.64 (0.42-0.97) | … |
| Years since T2DM diagnosis, years | | 1.04 (1.02-1.06) | … |
| Serum HbA1c, mmol/mol | | 1.01 (1.00-1.03) | … |
| Insulin use | | 1.27 (0.91-1.78) | … |
| eGFR <60 ml/min/1.73m^2^ | | 0.57 (0.38-0.85) | … |
| Albuminuria | | 1.91 (1.32-2.75) | 1.62 (1.02-2.57) |
| Retinopathy | | 1.84 (1.19-2.83) | … |
| Polyneuropathy | | 1.83 (1.29-2.60) | 1.69 (1.09-2.61) |
| Coronary heart disease | | 1.36 (0.91-2.04) | … |
| Cerebrovascular disease | | 1.94 (1.10-3.45) | … |
| Number of prebaseline hyperkalemia events | | |  |
|  | 0 | ref |  |
|  | 1-2 times | 0.70 (0.32-1.59) | … |
|  | 3-5 times | 0.60 (0.24-1.48) | … |
|  | >5 times | 1.07 (0.32-3.58) | … |
| Number of prebaseline acute kidney injury events | | 1.11 (0.79-1.54) | … |

*… excluded in the multivariate model in backward step analysis due to non-significance.*

**Appendix 1.** Maximal dosages of renin-angiotensin-aldosterone-system inhibitors.

Angiotensin converting enzyme inhibitors: captopril 150 mg, enalapril 40 mg, lisinopril 40 mg, perindopril 8 mg, ramipril 10 mg, quinapril 40 mg, benazepril 40 mg and fosinopril 40 mg. Angiotensin II receptor blockers (ARB): losartan 100 mg, eprosartan 600 mg, valsartan 320 mg, irbesartan 300 mg, candesartan 32 mg, telmisartan 80 mg and olmesartan 40 mg.
